# High-throughput phenotyping technologies allow accurate selection of stay-green

**DOI:** 10.1093/jxb/erw301

**Published:** 2016-09-06

**Authors:** Greg J. Rebetzke, Jose A. Jimenez-Berni, William D. Bovill, David M. Deery, Richard A. James

**Affiliations:** ^1^CSIRO Agriculture and Food, PO Box 1700, Canberra, ACT 2601, Australia; ^2^High Resolution Plant Phenomics Centre, Australian Plant Phenomics Facility, CSIRO Agriculture and Food, PO Box 1700, Canberra, ACT 2601, Australia

**Keywords:** Climate change, crop adaptation, crop breeding, drought, genotype ×, environment interaction, leaf senescence, phenotyping, stay-green, wheat

**Improved genotypic performance in water-limited environments relies on traits, like ‘stay-green’, that are robust and repeatable, correlate well across a broader range of target environments and are genetically more tractable than assessment of yield *per se*. Christopher *et al.* (see pages 5159–5172) used multi-temporal, Normalised Difference Vegetative Index (NDVI) measurements with crop simulation modelling to demonstrate the value of various stay-green phenotype parameters for improving grain yield across different environment types.**

Plant breeding is a slow and costly process. The potential release of a new, improved crop variety is limited by many factors, including the extent of genetic variation in key traits that contribute to yield, the confidence a breeder has in the selected phenotype and its association with underlying genotype, and the genotypic association of selected traits with extrapolation to performance across environments. Yield is phenotypically complex, reflecting an underlying genetic complexity and often unpredictable gene–environment interactions, and hence selection for less complex surrogates is desirable.

Selection for greater yield in water-limited and hot environments is particularly challenging owing to reduced heritability that reduces the breeders’ ability to identify elite families or lines. Frameworks reflecting underlying biological understanding of adaptation have been hypothesized and numerous traits suggested specifically targeting adaptation to target environments (e.g. Richards *et al.*, 2010). These traits should complement yield-based selection but allow enrichment in early generations prior to the more expensive multi-environment testing of elite lines for yield *per se*.

‘Stay-green’ is a measure of a genotype’s capacity to maintain carbon assimilation over an extended period. Maintenance of green leaf area has been demonstrated to sustain carbon assimilation during grain-filling (Thomas and Smart, 1993). Plants (more specifically ‘canopies’) respond dynamically to environmental stresses, but the extent of this change during grain-filling varies considerably depending on genotype (e.g. Thomas and Ougham, 2014). Differential maintenance of green leaf area through grain-filling has been associated with increased grain yield in wheat (Lopes and Reynolds, 2012; Christopher *et al.*, 2014) and maize (Trachsel *et al.*, 2016), while in the case of sorghum stay-green is a key trait targeted in breeding programmes (e.g. Borrell *et al.*, 2014). As demonstrated by Christopher *et al.* (2016), the value in stay-green across species is in improved genotypic adaptation to terminal drought. Importantly, the adaptation reported by Christopher and colleagues is likely to become even more relevant in future climates where air temperatures are predicted to increase through grain-filling (Asseng *et al.*, 2011).

## The challenge

Stay-green is in itself a broad phenotype. Christopher *et al.* (2016) and others (e.g. Thomas and Smart, 1993) describe stay-green as the dynamic in leaf greenness, which reflects both functional (underlying photosynthetic capacity) and non-functional, cosmetic characteristics. As a phenotype, stay-green can represent the underlying genotypic driver of assimilation, but can also simply reflect slowed water use, greater nitrogen uptake or slowed nitrogen remobilization, or any combination of these and potentially other physiological or developmental factors (Borrell *et al.*, 2001; Thomas and Howarth, 2000). A clear challenge is to separate cosmetic characteristics of ‘stay-green’ from functional characteristics.

Phenotyping of stay-green has relied historically upon the breeder’s eye. Initial phenotyping efforts focused on visual scores (Thomas and Smart, 1993), but in recent years spectral indices providing a quantitative basis for trait dissection have become more accessible. A typical surrogate for stay-green, Normalised Difference Vegetative Index (NDVI), can be assessed readily and cheaply across large breeding populations. In the case of Christopher *et al.* (2016), NDVI assessment was undertaken using a commercial Greenseeker^®^ at multiple times throughout grain-filling, and at multiple well-characterized sites to derive parameters associated with improved performance in relevant environment types.

The NDVI is the normalized ratio of the difference between reflected light in the red and near-infrared bands of the electromagnetic spectrum (Rouse *et al.*, 1974). Its principle is based on the fact that in healthy, living canopies most of the red light is absorbed by the photosynthetic pigments, while the near-infrared light is reflected as a result of the light scattering in leaf internal structure and canopy architecture. Physiologically these values reflect an integrated mixture of biomass (or leaf area) and leaf chlorophyll (Lukina *et al.*, 1999). While NDVI is broadly predictive of canopy greenness, relationships with leaf biomass, leaf area and nitrogen are not always predictive in field plots (Hansen and Schjoerring, 2003); these tend to work better at early stages of canopy growth, well before canopy closure (G. J. Rebetzke, unpublished data). NDVI is also influenced by the soil reflectance (Huete, 1988) and in the case of passive sensors, the solar angle and cloudiness can also be critical.

## The opportunity

Christopher *et al.* (2016) demonstrate genotypic variation in stay-green parameters derived from NDVI to be a robust predictor of grain yield in different environment types. However, extension to parameters that describe the dynamic change in leaf greenness and architecture throughout the canopy might allow changes in stay-green to be linked to changes in leaf area and remobilization of leaf nitrogen during grain-filling. They may also provide greater insight to breeders exploring genotypic variation for traits contributing to maintenance of green leaf area. Coupling of these measures with accurate assessment of canopy temperature should allow separation of functional from cosmetic stay-green.

A portable Phenomobile (Box 1, upper panel) developed at CSIRO by the High Resolution Plant Phenomics Centre (Deery *et al.*, 2014) incorporates a Greenseeker^®^ to reliably quantify NDVI (Box 1, lower panel) and LiDAR (from ‘Light Detection And Ranging’) to characterize the vertical distribution of green leaf biomass and leaf area within the canopy (Box 2). Using GPS-linked geo-referencing, one hectare of breeding lines (about 1000 plots) can be accurately and non-destructively assessed for canopy architecture characteristics in under an hour. Box 1 (lower panel) summarizes evolution in NDVI during grain-filling for 64 wheat genotypes contrasting in canopy architecture. All genotypes were sown under both controlled rainfed (water-limited) and irrigated conditions after Rebetzke *et al.* (2013*a*). Changes in NDVI were systematic until early- to mid-October when high temperatures impacted differentially on changes in leaf senescence [Box 1, lower panel (a)]. Changes in NDVI were repeatable across genotypes in both irrigated and rainfed environments [*cf.* C676+ vs C676–, Box 1, lower panel (b)].

**Box 1. The upper panel shows the portable Phenomobile Lite unit being deployed in the field. The lower panel shows the evolution of NDVI from pre-anthesis to maturity at Yanco (New South Wales, Australia) in 2015 for (a) a range of leaf architecture wheat near-isogenic lines (NILs) assessed under rainfed and irrigated conditions, and (b) two leaf-erectness NILs (planophile, C676+ and erect, C676–) in water-limited, rainfed (RF) and irrigated (Irr) conditions.**

**Figure:**
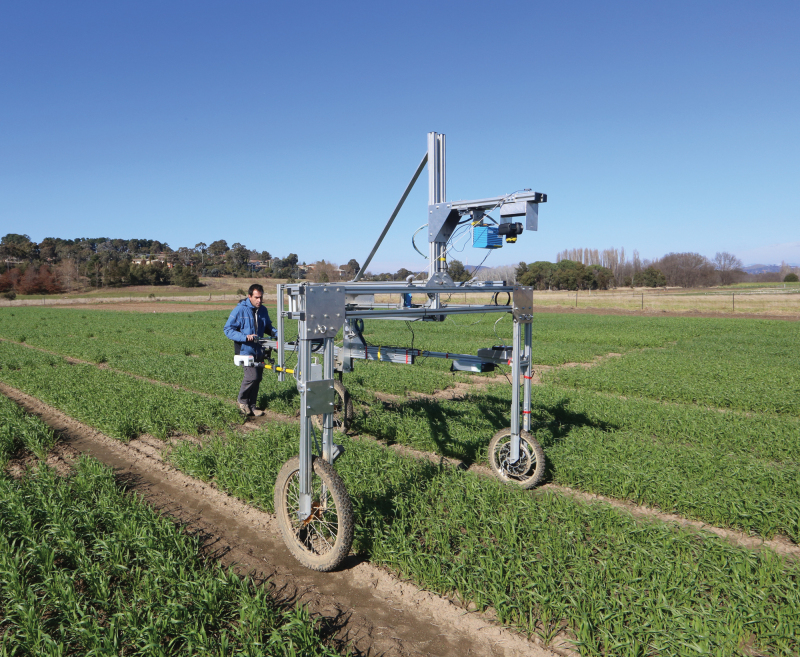

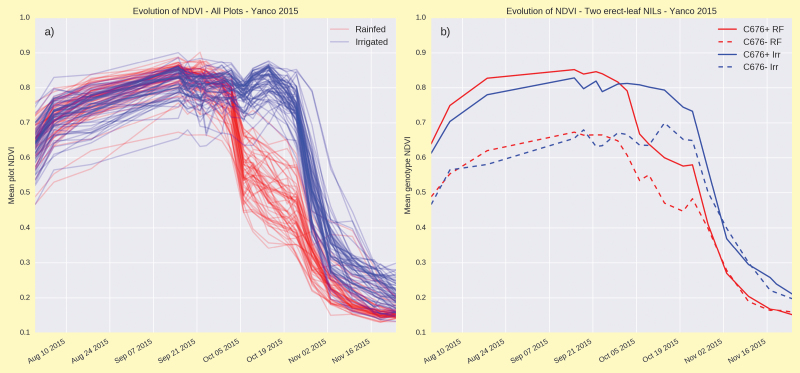

LiDAR incorporating a red laser captured the bi-weekly change in vertical distribution of the green vegetation profile (Box 2). The use of the red laser has the equivalence of the red channel in the NDVI with the green vegetation absorbing most of the red light. In turn, differences in the reflected signal from the laser provides information of canopy greenness. Since the LiDAR delivers a 3D point-cloud, it is possible to determine the distribution of green leaf area across the vertical profile within the canopy. Moreover, the 3D point-cloud reveals differences in canopy architecture and light interception (Box 2). Deployment of the Phenomobile on a regular basis during the development of the crop provides a dynamic insight into the timing, location and amount of leaf senescence across different genotypes, such as for leaf erectness near-isogenic lines (NILs) in Box 2. See also the animation at 10.6084/m9.figshare.3502865 [https://dx.doi.org/10.6084/m9.figshare.3502865] from before anthesis to maturity for the same two leaf erectness NILs; fractional (F) cover and NDVI scores are also given for each assessment date.

**Box 2. The upper panel shows canopy reconstruction from a LiDAR point cloud for two leaf erectness NILs (planophile, C676+ and erect, C676–), with the actual crop pictured alongside. The lower panel similarly shows canopy reconstruction, with evolution of canopy architecture and green leaf area distribution for the two leaf-erectness NILs in rainfed and irrigated conditions during grain-filling. Each plot shows the vertical canopy profile across 2–3 rows in each plot in the field. The colour is the relative absorption of the red light from the LiDAR, thus representing the greenness of the canopy (green and orange points are green and senescing leaves, respectively). See also the animation at 10.6084/m9.figshare.3502865 [https://dx.doi.org/10.6084/m9.figshare.3502865] from before anthesis to maturity for the same two leaf-erectness NILs; fractional (F) cover and NDVI scores are also given for each assessment date.**

**Figure:**
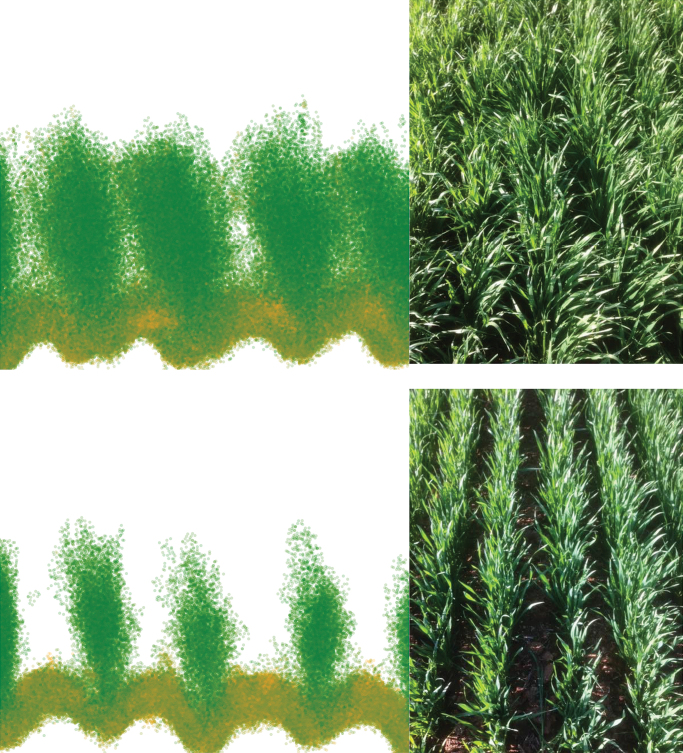

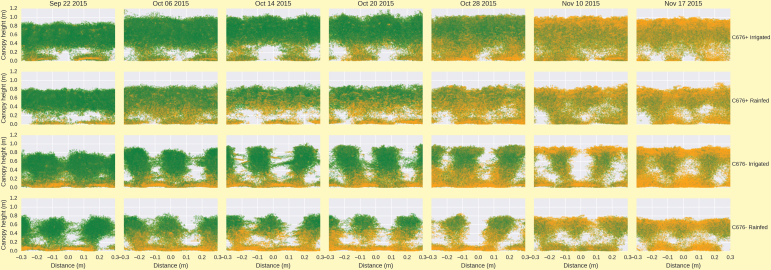

Assembling all bi-weekly LiDAR images together generates a temporal point-cloud detailing changes in canopy development for any genotype in a population. Box 3 compares canopy development throughout the season for two wheat NILs contrasting in leaf erectness. Differences in plant stature and canopy erectness are clearly demonstrated with maintained spacing between rows of plants for the erect NIL C676–, whereas canopy closure occurred early for the planophile leaf NIL C676+.

Cooler canopies infer greater stomatal conductance and potentially photosynthetic rates (Rebetzke *et al.*, 2013*b*); therefore measuring canopy temperature in conjunction with green leaf area distribution assessed with LiDAR may allow separation of functional from cosmetic stay-green. Cooler canopies were previously associated with increased stay-green across different sowing dates in a large and diverse wheat population (Kumari *et al.*, 2013). By deploying ArduCrop^®^ thermo-imaging sensors (Box 3, upper panel) in the canopy architecture experiment above, monitoring changes in canopy temperature every five minutes (Box 3, lower panel), we were able to confirm that NILs selected with canopy architectures as phenotypically erect, waxy or rolling were cooler at anthesis than their near-isogenic counterparts. These canopies remained cooler late into grain-filling with the exception of the relative warming of the erect canopy NILs (Box 3, lower panel). Throughout grain-filling, average reduction in canopy temperature was 0.29, 0.69 and 0.25 °C for erect, waxy and rolling leaf architecture NILs, respectively (data not shown). The greater individual costs of ArduCrop^®^ sensors will limit their use in breeding. However, simple extension to aerial thermo-imaging will allow many thousands of breeding lines to be reliably assessed for canopy temperature (D.M. Deery, unpublished data).

**Box 3. The upper panel shows the ArduCrop^®^ thermo-imaging sensor deployed in the field. Deviations in mean canopy temperature for leaf architecture isomorphic groups (‘roll’, 10 rolling vs non-rolling; ‘wax’, eight waxy vs non-waxy; ‘erect’, eight erect vs planophile flag and penultimate flag leaves) throughout the day early (left-hand panel) and late (right-hand panel) in grain-filling. Each hourly value represents averages across five-minute time intervals for each group of lines.**

**Figure:**
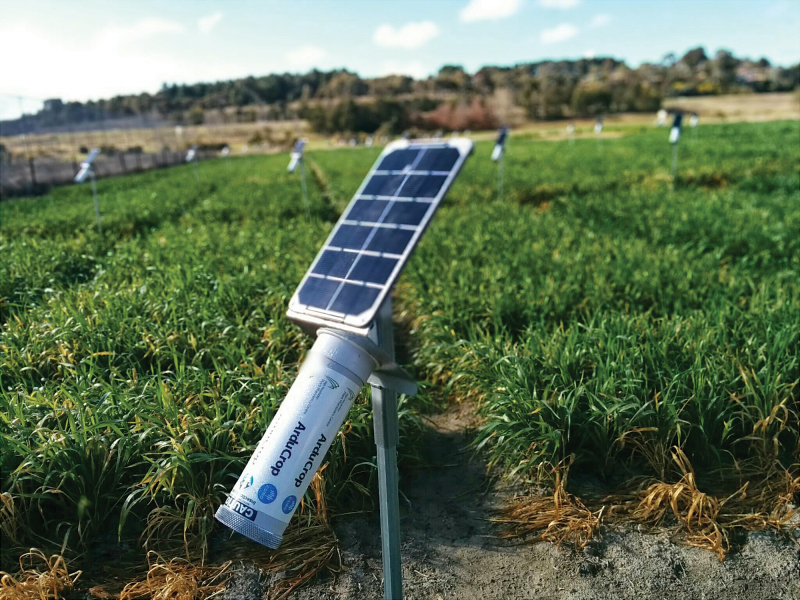

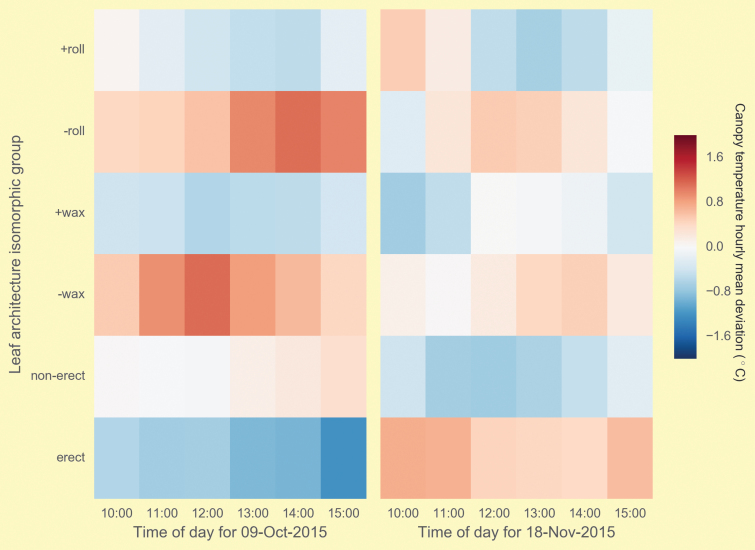

## Step-changes ahead

The work of Christopher *et al.* (2016) highlights how reliable phenotyping of stay-green together with environmental characterization and simulation can identify and then predict trait value for use in breeding. By complementing NDVI with new high-throughput phenotyping tools that have the capacity to carefully monitor changes in leaf area, greenness and photosynthetic capacity (via changes in canopy temperature) step-changes in selection of functionally stay-green germplasm from large breeding populations can be achieved.
